# Supplementary material for: Host Specificity and Preliminary Impact of Lepidapion argentatum (Coleoptera, Brentidae), a Biocontrol Candidate for French Broom (Genista monspessulana, Fabaceae)
Source: Insects. 2021 Jul 31;12(8):691. doi: 10.3390/insects12080691 (PMC8396517; doi:10.3390/insects12080691)
Supplement: Supplementary file 1 [file insects-12-00691-s001.zip › insects-1279845-supplementary.pdf]

Supplementary Materials: The following are available online at www.mdpi.com/xxx/s1,

Table S1. Non-target host plant list for host specificity testing of French broom (Akers 2005); in bold, test plant species used for host specificity tests in 2012 and/or 2015.

Family	Tribe	Species	A: annual / P: perennial	Origin	Seeds sources
Mimosaceae	*Acacieae*	***Acacia dealbata***	**P**	Australia	Australia
Mimosaceae	*Acacieae*	*Acacia melanoxylon*	P	Australia	Australia
Fabaceae	*Amorpheae*	*Amorpha canescens*	P	USA	USA
Fabaceae	*Amorpheae*	***Amorpha fruticosa***	P	USA	USA
Fabaceae	*Loteae*	*Anthyllis vulneraria*	A/P	North Atlantic	USA
Fabaceae	*Loteae*	*Anthyllis vulneraria*	A/P	North Atlantic	France
Fabaceae	*Galegeae*	***Astragalus canadensis***	P	North Atlantic	USA
Fabaceae	*Galegeae*	***Astragalus nuttalii***	P	California	USA
Fabaceae	*Galegeae*	***Astragalus trichopodus***	P	California	USA
Fabaceae	*Sophoreae*	***Baptisia australis***	P	USA	USA
Fabaceae	*Sophoreae*	***Baptisia bracteata***	P	USA	USA
Fabaceae	*Cladrastis clade*	***Cladrastis lutea***	P	USA	USA
Fabaceae	*Crotalarieae*	***Crotalaria sagittalis***	A/P	USA	USA
Fabaceae	*Genisteae*	***Cytisus racemosus***	P	Europe, Africa, Asia	CSIRO
Fabaceae	*Genisteae*	***Cytisus proliferus***	P	Canaries Island	CSIRO
Fabaceae	*Genisteae*	***Cytisus scoparius***	P	Europe	France
Fabaceae	*Genisteae*	***Cytisus striatus***	P	Iberian Peninsula	Oregon
Fabaceae	*Genisteae*	***Cytisus villosus***	P	Mediterranean basin	Spain
Fabaceae	*Genisteae*	***Genista linifolia***	P	Europe	Canaries Island
Fabaceae	*Genisteae*	***Genista monspessulana***	P	Mediterranean basin	USA
Fabaceae	*Genisteae*	***Genista monspessulana***	P	Mediterranean basin	France
Fabaceae	*Genisteae*	*Genista "porlock"*	P	Europe, Asia et Africa	commercial
Fabaceae	*Genisteae*	***Genista stenopetala***	P	Madeira, Canaries Island	Canaries Island
Fabaceae	*Phaseoleae*	***Glycine max***	A	Asia	commercial
Fabaceae	*Psoraleeae*	***Hoita macrostachya***	P	California	USA

Fabaceae	*Fabeae*	***Lathyrus vestitus***	P	USA	USA
Fabaceae	*Loteae*	***Lotus scoparius***	A	California	USA
Fabaceae	*Genisteae*	***Lupinus albus***	A	Mediterranean basin	Australia
Fabaceae	*Genisteae*	***Lupinus angustifolius***	A	Europe	Australia
Fabaceae	*Genisteae*	***Lupinus atlanticus cosentini***	A	Mediterranean basin	CSIRO
Fabaceae	*Genisteae*	***Lupinus arboreus blue***	P	California	CDFA
Fabaceae	*Genisteae*	***Lupinus chamissonis***	A	California	USA
Fabaceae	*Genisteae*	***Lupinus cosentini***	A	Mediterranean basin	CSIRO
Fabaceae	*Genisteae*	***Lupinus formosus***	A	California and Oregon	USA
Fabaceae	*Genisteae*	***Lupinus luteus***	A	Mediterranean basin	Australia
Fabaceae	*Genisteae*	***Lupinus microcarpus densiflorus***	A	USA	CDFA
Fabaceae	*Genisteae*	*Lupinus perennis*	P	USA	USA
Fabaceae	*Genisteae*	***Lupinus pilosus***	A	Israel	Australia
Fabaceae	*Genisteae*	***Lupinus mutabilis***	A	South-West America (Andean region)	CSIRO
Fabaceae	*Genisteae*	***Lupinus texensis***	A	Texas	USA
Fabaceae	*Phaseoleae*	*Phaseolus vulgaris*	A	Central and South America	commercial
Fabaceae	*Thermopsideae*	*Pickeringia montana*	P	California	USA
Fabaceae	*Fabeae*	*Pisum sativum*	A	Mediterranean basin	commercial
Fabaceae	*Sesbanieae*	***Sesbania exaltata***	A	USA	USA
Fabaceae	*Sophoreae*	*Sophora secundiflora*	P	South America	New Mexico
Fabaceae	*Genisteae*	***Spartium junceum***	P	Europe, Asia, Africa	USA
Fabaceae	*Sophoreae*	***Thermopsis macrophylla***	P	California	USA
Fabaceae	*Sophoreae*	***Thermopsis montana***	P	USA	USA
Fabaceae	*Trifolieae*	*Trifolium repens*	P	Europe, Asia, Africa and North America	commercial
Fabaceae	*Fabeae*	***Vicia faba***	A	Asia	commercial
